#  ‎ Association Between Neuregulin-1 Gene Variant ‎‎(rs2439272) and Schizophrenia and Its Negative ‎Symptoms in an Iranian Population

**Published:** 2016-07

**Authors:** Sadegh Yoosefee, Esmaeil Shahsavand Ananloo, Mohammad-Taghi Joghataei, Morteza Karimipour, Mahmoudreza Hadjighassem, Hoorie Mohaghghegh, Mehdi Tehrani-Doost, Amir-Abbas Rahimi, Hamid Mostafavi Abdolmaleky, Maryam Hatami

**Affiliations:** 1 Neuroscience and Neurology Research Center, Qom University of Medical Sciences, Qom, ‎Iran.; 2 Department of Neuroscience, School of Advanced Technologies in Medicine, Iran ‎University of Medical Sciences, Tehran, Iran‎.; 3 Health and Religion Research Center, Qom University of Medical Sciences, Qom, Iran.; 4 ‎Department of Adult Psychiatry, Roozbeh Hospital, School of Medicine, Tehran University of ‎Medical Sciences, Tehran, Iran.; 5 ‎Department of Genomic Psychiatry and Behavioral Genomics, Roozbeh ‎Psychiatry Hospital, School of ‎Medicine, Tehran University of Medical Sciences, Tehran, Iran.; 6 Molecular Medicine Group, Pasteur Institute of Iran, Tehran, Iran.; 7 Department of Neuroscience and Addiction Studies, School of Advanced Technologies in Medicine, Tehran ‎University of Medical ‎Sciences, Tehran, Iran.; 8 Department of Child and Adolescent Psychiatry, Roozbeh Hospital, School of Medicine, ‎Tehran University of Medical Sciences, Tehran, Iran.; 9 Department of Genetics & Genomics, Boston University School of Medicine, Boston, MA, ‎United States.

**Keywords:** Negative Symptoms, Neuregulin-1 (NRG1), Positive and Negative Syndrome Scale ‎‎ (PANSS), Schizophrenia, Single Nucleotide Polymorphism (SNP)

## Abstract

**Objective: **Although the etiology of schizophrenia is unknown, it has a significant genetic component. ‎A number of studies have indicated that neuregulin-1 (NRG1) gene may play a role in the ‎pathogenesis of schizophrenia. In this study, we examined whether the rs2439272 of NRG1 ‎is associated with schizophrenia and its negative symptoms in an Iranian population.‎

**Method: **Rs2439272 was genotyped in 469 participants including 276 unrelated patients with schizophrenia and 193 healthy controls. The association of genetic risk with negative ‎symptoms (by using panss) was examined in the total, male and female samples. COCAPHASE and ‎CLUMP22 programs were used to compare the allele and genotype frequencies, and ‎general linear regression was used to analyze the quantitative dependent variables by the ‎selected variant.‎

**Results: **In this study, it was revealed that the G allele of rs2439272 might be an allele with the ‎increased risk of developing schizophrenia, especially in the male participants. In addition, ‎significant differences were found between the G allele and GG genotype frequencies, and negative symptoms in the total and male participants.‎

**Conclusion: **Our results supported the association between rs2439272 in NRG1 gene and risk of ‎schizophrenia and its negative symptoms in an Iranian population. ‎

Schizophrenia (OMIM: 181500) is a severe mental disorder, afflicting 1% of the world’s ‎population, and is characterized by positive, negative, disorganized and cognitive ‎symptoms. Although its etiology is unknown, schizophrenia has a significant genetic ‎component. A number of studies have indicated that 8p22–p12 is likely to harbor ‎schizophrenia susceptibility locus (1-3). In this region, the candidate gene of interest, ‎NRG1, that is involved both in neurodevelopment and neurotransmitter mechanisms in ‎the brain, may play a role in the pathogenesis of schizophrenia. Stefansson et al. (4) ‎reported the over-representation of at-risk haplotype (“HapICE”) constructed from five ‎SNPs and two microsatellite markers in NRG1 gene for schizophrenia in the Icelandic ‎population. Other research groups found different haplotypes in different ethnic groups ‎‎ (5-7). Recently, it is reported that rs2439272 of NRG1 has significant association across ‎working memory domains in schizophrenic patients in Asian populations (8). The aim of this ‎study was to gain insight into the role of selected SNP and schizophrenia and its symptoms ‎in an Iranian population. ‎‎

## Materials and Method

***Participants***

Four hundred sixty nine Iranian participants, including 276 unrelated patients with ‎schizophrenia and 193 healthy matched controls, were evaluated. Schizophrenia diagnosis ‎was determined independently by two expert psychiatrists according to the DSM-IV-TR ‎criteria. Written informed consent was obtained from all participants. This study was ‎approved by the Ethics Committees of Tehran University of Medical Sciences, Tehran, Iran. ‎Demographic features are shown in Table 1.‎

***DNA Preparation, SNPs Selection and Genotyping***

**Figure1:**
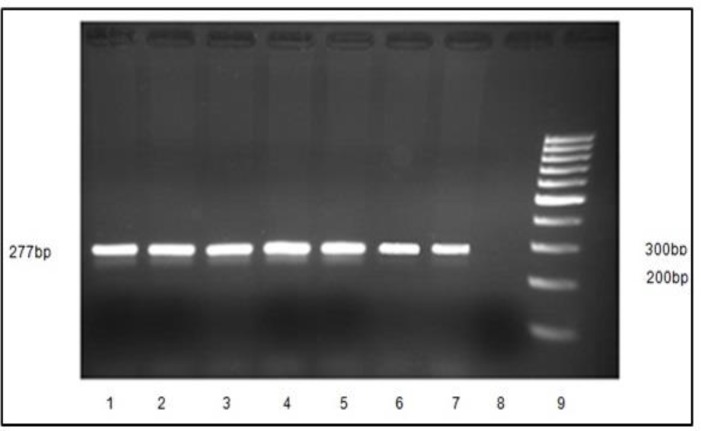
Electrophoresis pattern for the PCR

**Figure2:**
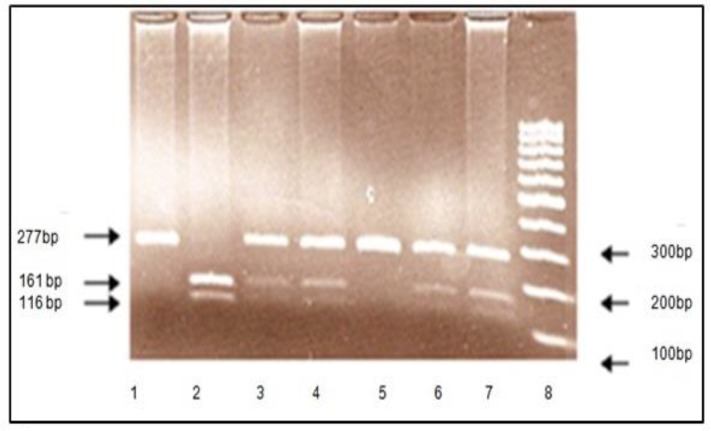
The RFLP pattern of RsaI ‎restriction ‎enzyme ‎digestion. 1: Undigested, 2: ‎homozygous for T/T, ‎‎3, 4, 6, 7: heterozygote ‎for ‎both T/C, 5: homozygous for ‎CC.

All blood samples were taken by vacuum tube pre-ﬁlled with the anticoagulant EDTA. High ‎molecular weight genomic DNA was prepared from venous blood using the salting out ‎procedure (9). The SNP rs2439272 was selected using the related literature and databases ‎‎ (e.g., UCSC Genome Browser; http://genome.ucsc.edu/, SNPper; http://snpper.chip.org/, ‎and HapMap; http://hapmap.ncbi.nlm.nih.gov/). Genotyping was performed blind to ‎status using polymerase chain reaction (PCR), and restriction fragment length ‎polymorphism-PCR (RFLP-PCR). Primers were designed using the Primer3 ‎‎ (http://frodo.wi.mit.edu/cgi-bin/primer3/primer3_www.cgi). The primers were used in the ‎reactions had the following sequences: Forward: 5′- TTG GCA ATG CAA AAG AAT AG -3′; ‎reverse: 5′- ACA GCA CAT TTC CTG ATC AG -3′. Annealing temperature and PCR product ‎size were 56ºC and 277 bp, respectively (Figure 1). Restriction enzyme was RsaI and ‎corresponding restriction 

fragment sizes were 161 bp and 116 bp (Figure 2). Number of ‎genotypes callable was 100%, and minor allele frequency was greater than 2%. The ‎digested fragments were fractionated on 2% agarose gel. Furthermore, a random group of ‎samples was also re-genotyped by direct sequencing to conﬁrm the genotyping results of ‎restriction fragment enzyme method.‎

***Statistical Analysis***

‎The Hardy–Weinberg equilibrium for genotypic distributions in the two populations was ‎tested using the χ2 goodness-of-fit test. The allelic association was estimated using the ‎COCAPHASED (UNPHASED) program (10). The genotype frequencies between cases and ‎controls were compared using the CLUMP22 software (11) by running 1000 Monte Carlo ‎simulations. Genotypic association was tested by the χ2, or by Fisher’s exact test to assess ‎the significance of our results. Independent-samples t-test procedure was used to ‎compare the means of the quantitative test scores for both groups. General linear ‎regression model (Univariate) was utilized to analyze a quantitative dependent variable by ‎a single independent variable (NRG1 SNP). The significance level for all statistical tests was ‎set at P<0.05. ‎

## Results

***Clinical Assessments***

‎We assessed the psychopathology using the PANSS; all scores (including positive ‎symptoms, negative symptoms, general psychopathology and total score) were increased ‎significantly in case vs. control groups (P<0.001) (Table 2).‎

***Marker Analysis for Schizophrenia***

‎We compared the allele and genotype frequencies in cases and controls. Using ‎COCAPHASE, we found significant differences in allele frequencies between cases and ‎controls in total (χ² = 6.711, P = 0.009), and in male (χ² = 5.483, P = 0.019) participants (Table ‎‎3). ‎

Using CLUMP22, we found significant differences in GG genotype frequencies between ‎cases (0.26) and controls (0.16) in total (χ² = 7.730, P = 0.005), and in male participants (0.27 ‎in cases vs. 0.15 in controls; χ² = 6.273, P = 0.012). No significant differences were detected ‎in female participants (Table 4).‎

***Marker Analysis for the PANSS-negative Symptoms***

Our study showed significant differences between cases and controls for negative ‎symptoms in total (F = 3.043, P = 0.029), and male (F = 3.086, P = 0.047) participants. No ‎significant differences were detected in female patients (Table 5).‎

**Table1:** Demographic Characteristics of Case and Control Groups

**Characteristic**	**Case** **(n = 276)**	**Control** **(n = 193)**	**Statistics** **F(P value)**
**Sex**			
Male, N0 (%)‎	166 (60%)	122 (63%)	
Female, No (%)‎	110 (40%)	71 (37%)	
**Age (in years)**			
**Total**			
Years (Mean ± SD)	37.32 ± 10.58	37.63 ± 12.32	6.03 (0.83)
Range, years	18 - 70	18 - 75	
**Male**			
Years (Mean ± SD)	36.45 ± 10.60	39.23 ± 12.35	2.45 (0.13)
Range, years	18 - 70	18 - 75	
**Female**			
Age, years (Mean ± SD)	38.76 ± 10.51	34.67 ± 11.81	2.25 (0.08)
Range, years	20-67	19 - 60	
**Height (in centimeter)**			
**Total ** (Mean ± SD)	166.59 ± 9.47	168.80 ± 7.60	4.77 (0.14)
**Male **(Mean ± SD)	171.34 ± 7.62	171.59 ± 6.02	2.39 (0.87)
**Female **(Mean ± SD)	157.65 ± 5.15	161.50 ± 6.41	0.23 (0.05)
**Handedness**			
**Total**			
Right handed‎	‎0.87	‎0.88‎	
Left Handed	0.13	0.12	
**‎** **Male**			
Right handed	0.91	0.85	
Left Handed	0.09	0.15	
**Female**			
Right handed	0.81	0.90	
Left Handed	0.19	0.10	

**Table2:** Clinical Assessments of psychopathology between case and control groups by using the PANSS

**Clinical Assessments (Symptoms)**	**Case** **(n = 276)**	**Control** **(n = 193)**	**Statistics** **F (P value)**
**Total**			
PS^	24.46 ± 4.47	8.04 ± 0.80	57.05 (<0.001*)
NS^^	24.47 ± 5.09	8.31 ± 0.97	46.69 (<0.001*)
GPS^^^	46.40 ± 6.51	24.12 ± 3.07	13.75 (<0.001*)
TS^^^^	95.37 ± 11.58	40.32 ± 3.63	30.99 (<0.001*)
**Male**			
PS^	25.02 ± 4.42	8.11 ± 0.91	33.59 (<0.001*)
NS^^	24.72 ± 4.99	8.29 ± 1.04	32.12 (<0.001*)
GPS^^^	46.86 ± 6.68	24.22 ± 3.18	8.88 (<0.001*)
TS^^^^	96.67 ± 11.40	40.54 ± 3.78	17.08 (<0.001*)
**Female**			
PS^	23.62 ± 4.43	7.73 ± 0.54	30.50 (<0.001*)
NS^^	24.10 ± 5.25	8.35 ± 0.85	15.76 (<0.001*)
GPS^^^	45.70 ± 6.22	23.97 ± 2.90	6.50 (<0.001*)
TS^^^^	93.42 ± 11.63	39.93 ± 3.34	17.15 (<0.001*)

^ Positive symptoms

^^ Negative symptoms

^^^ General psychopathology symptoms

^^^^ Total score

* Significant

**Table3:** Allele frequencies of NRG1 SNP (rs2439272) among cases and controls using COCAPHASE

**Alleles**	**Case (number, %)**	**Control (number, %)**	**OR**	**95%CI**	**χ²**	***P value***
**Total (Cases [N=276]; Controls [N=193])**						
A	259 (47)	108 (56)				
G	293 (53)	85 (44)	1.412	1.087-1.834	6.711	0.009*
**Male (Cases [N=166]; Controls [N=122])**						
A	155 (47)	138 (57)				
G	177 (53)	108 (43)	1.488	1.066-2.073	5.483	0.019*
**Female (Cases [N=110]; Controls [N=71])**						
A	105 (48)	77 (54)				
G	115 (52)	65 (46)	1.297	0.850-1.981	1.485	0.23

* Significant

**Table4:** Genotype frequencies of NRG1 SNP (rs2439272) among cases and controls using CLUMP22

**Alleles**	**Case (number, %)**	**Control (number, %)**	**OR**	**95%CI**	**χ²**	***P value***
**Total (Cases [N=276]; Controls [N=193])**						
AA	50 (20)	52 (27)				
GA	150 (54)	111 (57)	1.278	0.813 - 2.007	1.130	**0.29**
GG	71 (26)	30 (16)	2.238	1.264 - 3.960	7.730	**0.005** *
GA + GG	221 (80)	141 (73)	1.511	0.938 - 2.431	3.168	**0.075** **
**Male (Cases [N=166]; Controls [N=122])**						
AA	34 (19)	34 (28)				
GA	87 (52)	70 (57)	1.243	0.703 - 2.198	0.560	**0.45**
GG	45 (27)	18 (15)	2.500	1.212 - 5.159	6.273	**0.012** *
GA + GG	132 (79)	88 (72)	1.500	0.869 - 2.591	2.127	**0.145**
**Female (Cases [N=110]; Controls [N=71])**						
AA	21 (22)	18 (25)				
GA	63 (57)	41 (58)	1.317	0.627 - 2.767	0.530	**0.47**
GG	26 (24)	12 (17)	1.857	0.733 - 4.705	1.719	**0.190**
GA + GG	89 (81)	53 (75)	1.439	0.704 - 2.944	1.001	**0.317**

* Significant

** Trend towards the significant effect

**Table5:** The effect of the rs2439272 on PANSS negative test scores in cases and controls: A general linear model analysis

**Subjects**	**PANSS-negative (Mean±SD)**	**PANSS-negative (Mean±SD)**	**F**	**P value**
	**Case**	**Control**		
**Total**	24.47 ± 5.09	8.31 ± 0.97	3.043	**0.029** *
**Male**	24.72 ± 4.99	8.29 ± 1.04	3.086	**0.047** *
**Female**	24.10 ± 5.25	8.35 ± 0.85	1.135	**0.32**

* Significant

## Discussion

In this study, we examined the association between the NRG1 gene selected SNP ‎‎ (rs2439272) and schizophrenia and its negative symptoms in a sample of 469 participants, ‎including 276 patients eith schizophrenia and 193 normal controls.‎

We found that the rs2439272 has a significant effect on the risk of developing ‎schizophrenia. This effect was found in the total and male samples. Furthermore, our ‎study results revealed that the G allele of the rs2439272, which is the major allele, might be ‎an allele with increased risk of developing schizophrenia, especially in male patients. 

Roussos et al. (12) reported the association between rs2439272 (G allele) and prepulse ‎inhibition (PPI) of the acoustic startle reflex, as a well validated ‎schizophrenia endophenotype. However, we could not find any studies between ‎rs2439272 and schizophrenia. Mohammad Shariati et al (3). in their study, showed a significant ‎association between schizophrenia and SNP8NRG241930 of NRG1 (located at the 5′ end of ‎this gene) in an Iranian population (3). Most studies have found an association between ‎schizophrenia and alleles that produce silent changes or occur in non-coding regions. It has ‎been shown that these polymorphisms (including SNP rs2439272 in our study that are ‎located in intronic region), affect NRG1 gene expression. For example, the low expression ‎of NRG1 messenger ribonucleic acid (mRNA) have been found in the brain of patients with schizophrenia (13, 14).‎

Neuregulin-1 gene is involved in different neurodevelopmental processes (15). It was ‎shown that mutations in NRG1 or ErbB (NRG1 receptor) genes induce a reduction in the ‎number of interneurons in the cortex (16) and hippocampal tissue (17). This gene is ‎necessary for establishing excitatory synapses in GABAergic interneurons and developing ‎balanced excitatory/inhibitory tone in the brain (18, 19). Therefore, it can be hypothesized ‎that there is a disturbance between excitatory and inhibitory factors, and negative ‎symptoms. The association between NRG1 and superior temporal gyrus (STG) anatomy ‎was explored in patients with schizophrenia. It was reported that the right posterior STG ‎had a significant positive correlation with negative symptoms (20). In their study, Tosato et ‎al. (21) suggested that NRG1 may be involved in determining STG size in schizophrenia and ‎may be associated with negative symptoms. According to the sex specific findings ‎between cases and controls, it has been suggested that the role of NRG1 may be sex ‎specific in regulating some behaviors (22). In animal studies, Karatsoreos et al (23). showed the ‎role of ovarian hormones to affect corticostrone response to stressors. Estrogen has also ‎been shown to modulate both excitatory and inhibitory states in neurons (23). It is possible ‎that the circulating gonadal hormones modulate NRG1-induced changes in GABAergic ‎neurotransmission during the development and adulthood to produce some of these sex-‎specific findings.‎

Using the PANSS, we found a significant effect of the SNP rs2439272 on negative ‎symptoms especially in male patients. Low expression of NRG1 mRNA have been found in ‎the brain of patients with schizophrenia (13, 14). In a functional study, Zhang et al (24). reported an ‎association between the level of NRG1 mRNA and PANSS ‎scores in a sample of schizophrenic patients. Only few studies assessed the association ‎between the specific NRG1 variants and PANSS test scores in patients with schizophrenia ‎‎[e.g., SNP8NRG241930 with the PANSS cognitive and hostility/excitability scores (25)].‎

## Limitations

There are certain limitations in the present study that should be acknowledged. First, the control sample was smaller in number than the case sample (193 vs. 276). However, our findings could be related to the relatively small sample size, so we cannot rule out the possibility of false negative findings. Our findings suggest that the GG genotype could have increased the risk of schizophrenia more than the AA or GA genotypes, especially in males. However, we recognize that the small sample size in our study limited the ability to draw more solid conclusions. Second, is the possible diversity in genetic backgrounds of the participants. So, the population substructure as a potential source for the association could be another limitation in analyses. Third, 60% of cases but 63% of controls were male, which might have an impact on gender-specific analyses. 

The scope of this study does not permit a detailed functional evaluation of the associated SNP. However, the future studies are needed to validate the role of the NRG1 selected SNP in schizophrenia and its psychopathology.

## Conclusion

‎For the first time, we showed that the NRG1 SNP (rs2439272 [A/G]) is significantly ‎associated with the risk of schizophrenia in an Iranian population. Moreover, our results ‎indicated that this SNP is associated with negative symptoms. These findings are consistent ‎with the theory that indicates NRG-1 gene variants may mediate risks for schizophrenia ‎and its negative symptoms.‎

## Databases

http://biotools.umassmed.edu/bioapps/primer3_www.cgi/‎

http://frodo.wi.mit.edu/cgibin/primer3/primer3_www.cgi/

http://genome.ucsc.edu/‎

http://hapmap.ncbi.nlm.nih.gov/‎

http://pngu.mgh.harvard.edu/_purcell/gpc/‎

http://snpper.chip.org/‎

http://www.omim.org/‎
